# Supplementary material for: Characterization of the Hoof Bacterial Communities of Active Digital Dermatitis Lesions in Feedlot Cattle
Source: Microorganisms. 2024 Jul 19;12(7):1470. doi: 10.3390/microorganisms12071470 (PMC11278616; doi:10.3390/microorganisms12071470)
Supplement: Supplementary file 1 [file microorganisms-12-01470-s001.zip › microorganisms-3051131-supplementary.pdf]

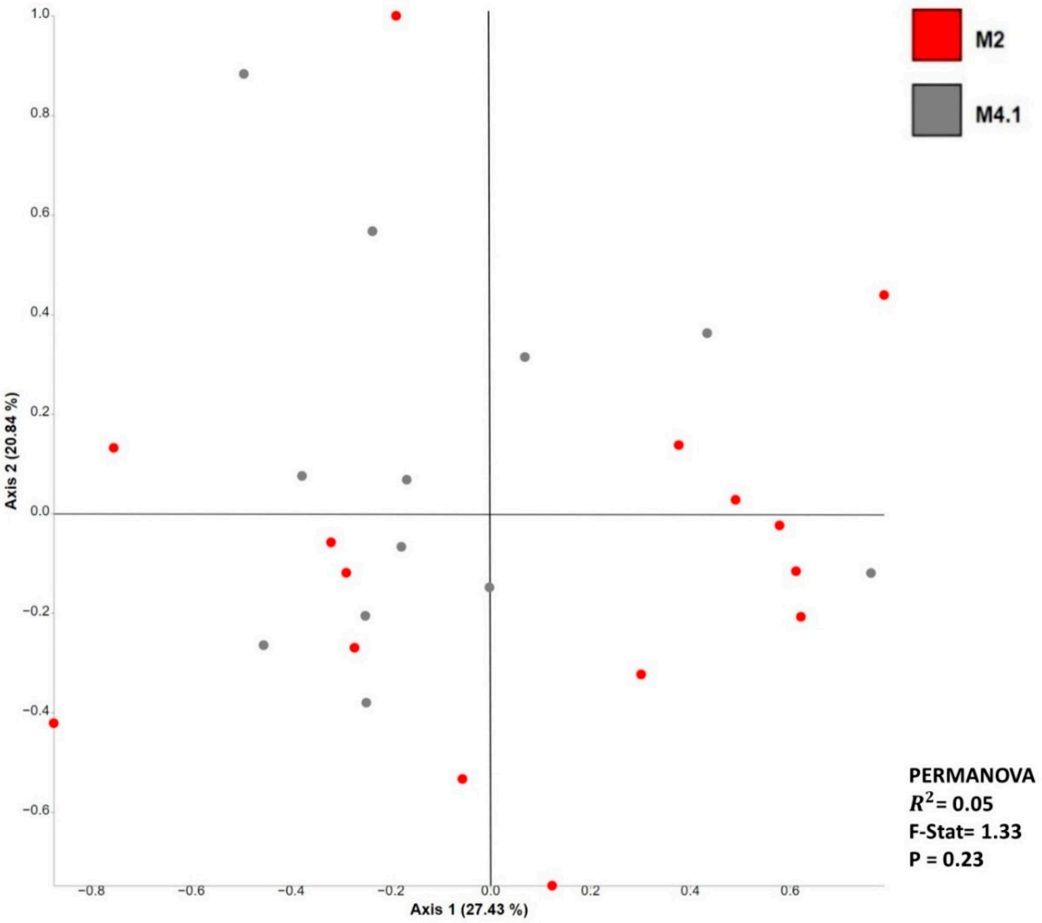

Figure S1. Principle coordinates analysis plot of weighted Unifrac distance metric generated from hoof bacterial communities comparison of hoof bacterial profiles generated from M2 and M4.1 stage.

Table S1. Sequence quality control of samples by DADA2 of study samples.

Sample-id	Final category	M-stage	Input	Filtered	Percentage of input passed filter	Denoised	Merged	Percentage of input merged	Non-chimeric	Percentage of input non-chimeric
S105	DD LESION	M2	65059	49891	76.69	48396	41086	63.15	19680	30.25
S106	DD CONTROL	M0	61411	48071	78.28	45525	34115	55.55	23138	37.68
S129	DD LESION	M2	63093	49812	78.95	48474	40592	64.34	12589	19.95
S130	DD CONTROL	M0	63058	49286	78.16	46501	31442	49.86	19082	30.26
S131	DD CONTROL	M0	65489	51317	78.36	48594	34910	53.31	20187	30.83
S132	DD LESION	M2	51595	35960	69.7	34539	29404	56.99	9487	18.39
S133	DD CONTROL	M0	44404	36275	81.69	34523	23364	52.62	12648	28.48
S134	DD LESION	M2	50102	38625	77.09	36898	30916	61.71	10727	21.41
S135	DD LESION	M4.1	56890	39919	70.17	38480	33424	58.75	11702	20.57
S136	DD CONTROL	M0	47268	38001	80.39	36075	25261	53.44	13358	28.26
S137	DD LESION	M4.1	67007	52329	78.09	50121	37290	55.65	21589	32.22
S138	DD LESION	M4.1	60510	46142	76.26	44276	36464	60.26	16987	28.07
S149	DD LESION	M2	57958	43986	75.89	43198	37609	64.89	4371	7.54
S158	DD CONTROL	M0	63695	46561	73.1	44250	34694	54.47	15424	24.22
S160	DD LESION	M2	35870	25782	71.88	25187	20779	57.93	5492	15.31
S169	DD CONTROL	M0	59790	50050	83.71	44346	30675	51.3	13854	23.17
S171	DD LESION	M4.1	59791	48949	81.87	47399	41519	69.44	13674	22.87
S172	DD CONTROL	M0	51158	38460	75.18	36910	29845	58.34	16204	31.67
S174	DD LESION	M2	62374	39908	63.98	39155	33104	53.07	7931	12.72
S175	DD CONTROL	M0	59841	47961	80.15	46112	35336	59.05	20616	34.45
S176	DD LESION	M2	56519	41387	73.23	39620	31368	55.5	11918	21.09
S177	DD LESION	M2	66050	52683	79.76	51610	45522	68.92	10701	16.2
S178	DD CONTROL	M0	65197	51203	78.54	48748	35016	53.71	18911	29.01
S179	DD LESION	M2	58605	40148	68.51	38793	34709	59.23	10696	18.25
S18	DD CONTROL	M0	58078	46283	79.69	43882	33162	57.1	20554	35.39
S180	DD CONTROL	M0	57620	45788	79.47	43645	32624	56.62	20504	35.58
S181	DD LESION	M4.1	68838	54163	78.68	51840	42019	61.04	17941	26.06
S182	DD LESION	M4.1	69011	53168	77.04	51789	44417	64.36	12270	17.78
S184	DD CONTROL	M0	58206	45838	78.75	44309	34354	59.02	17740	30.48
S186	DD LESION	M2	57696	38664	67.01	37699	33507	58.08	10184	17.65
S187	DD CONTROL	M0	50437	40026	79.36	38045	27787	55.09	15759	31.24
S189	DD LESION	M2	68707	46375	67.5	44233	37478	54.55	10932	15.91
S19	DD LESION	M4.1	60190	45266	75.21	44292	37416	62.16	10324	17.15
S190	DD CONTROL	M0	64176	50473	78.65	47840	36505	56.88	22515	35.08
S192	DD LESION	M4.1	48007	27226	56.71	26519	20256	42.19	6914	14.4
S193	DD LESION	M2	39809	29903	75.12	29463	26556	66.71	9127	22.93
S194	DD CONTROL	M0	42368	30722	72.51	28749	21575	50.92	12841	30.31

S195	DD CONTROL	M0	45037	34053	75.61	32008	22219	49.33	18029	40.03
S196	DD LESION	M2	55066	36580	66.43	35454	31168	56.6	8977	16.3
S197	DD LESION	M4.1	40972	32502	79.33	31603	28181	68.78	9694	23.66
S198	DD CONTROL	M0	52981	42132	79.52	39769	27041	51.04	15235	28.76
S199	DD CONTROL	M0	54856	41842	76.28	39649	25471	46.43	14362	26.18
S200	DD LESION	M4.1	54140	39466	72.9	37759	30449	56.24	11959	22.09
S201	DD LESION	M4.1	61916	45824	74.01	45208	40397	65.24	11091	17.91
S202	DD LESION	M4.1	53373	38771	72.64	36911	30868	57.83	15761	29.53
S203	DD CONTROL	M0	52571	42692	81.21	39120	25585	48.67	15774	30.01
S216	DD CONTROL	M0	44628	33268	74.55	32154	26144	58.58	13976	31.32
S217	DD LESION	M2	43737	33073	75.62	32656	29715	67.94	6949	15.89
S226	HC CONTROL	M0	45470	36120	79.44	34726	28406	62.47	11776	25.9
S227	HC CONTROL	M0	47613	36724	77.13	34852	27808	58.4	15679	32.93
S228	HC CONTROL	M0	57912	43738	75.52	41586	31972	55.21	19190	33.14
S229	HC CONTROL	M0	44057	33981	77.13	31911	24405	55.39	15851	35.98
S230	HC CONTROL	M0	58033	43449	74.87	41310	31550	54.37	19806	34.13
S231	HC CONTROL	M0	40502	32215	79.54	30234	22022	54.37	13975	34.5
